# Necroptosis-Related Modification Patterns Depict the Tumor Microenvironment, Redox Stress Landscape, and Prognosis of Ovarian Cancer

**DOI:** 10.1155/2023/4945288

**Published:** 2023-04-11

**Authors:** Rui Geng, Zihang Zhong, Senmiao Ni, Wen Liu, Zhiqiang He, Shilin Gan, Qinghao Huang, Hao Yu, Jianling Bai, Jinhui Liu

**Affiliations:** ^1^Department of Biostatistics, School of Public Health, Nanjing Medical University, 101 Longmian Avenue, Jiangning District, Nanjing 211166, China; ^2^Department of Gynecology, The First Affiliated Hospital of Nanjing Medical University, Nanjing, 210029 Jiangsu, China

## Abstract

Necroptosis is one of programmed cell death discovered recently, which involves in tumorigenesis, cancer metastasis, and immune reaction. We studied the necroptosis-related genes (NRGs) in ovarian cancer (OV) tissues using data from public databases, which separated into two NRGclusters. Patients in cluster A would have severe clinical characteristics, poor prognosis, and worse tumor microenvironment infiltration characteristics. The NRG score was achieved through the Cox analysis, along with a construction of a prognostic model. People with lower risk score would have better prognosis, lower expression of redox related genes, higher immunogenicity, and better effect on immunotherapy. In addition, the NRG score was closely related to cancer stem cell index, copy number variations, tumor mutation load, and chemosensitivity. We built a nomogram to enhance clinical application of the signature. These outcomes can help use know the function of NRGs in OV and provide new ideas for evaluating clinical outcome and developing more effective treatment protocols.

## 1. Introduction

Ovarian cancer (OV) is a major gynecological malignancy around the world, and its mortality ranks first among gynecological malignancies [1]. Worldwide, the number of new OV cases was 313,959 and the number of deaths was 207,252 in 2020 [2]. Although the treatment of OV has made many progresses recently, the prognosis of OV is still not well [3]. More than 60% patients were in an advanced stage when diagnosed [4, 5]. Through timely diagnosis and appropriate treatment, the mortality of advanced stage and recurrence rate of OV can be reduced to great extent [6]. So, it is needed to found new diagnostic and therapeutic methods. With development for the branch of the cancer cell immune recognition and immune regulate molecules, immunotherapy has become a research hotspot recently [7]. Developing new biomarkers, identifying therapeutic targets, predicting therapeutic effects, and screening potential immunotherapeutic drugs offer new orientation for the remedy of OV and may prolong the survival of patients [8].

Necroptosis is defined as a regulated type of necrosis whose morphology is similar to necrosis, such as cell swelling and rupture, regulated by certain signal pathways like apoptosis [9]. Necroptosis is crucial to cancer. For one thing, necroptosis can trigger adaptive immune response and impede tumor progression [10]. Meanwhile, the inflammatory response may also help the occurrence and development of cancer, and necroptosis can produce immunosuppressive tumor microenvironment (TME), which may contribute to cancer progression [11, 12]. Until now, several chemotherapeutics, natural compounds, and classical necroptosis inducers have been proved to disappear tumor cells through necroptosis. For instance, characteristics such as etoposide, 5-FU, and cisplatin may lead necroptosis of cancer cells [13]. Consequently, the selection of necroptosis-related genes and using them to build predict signatures were promising methods to forecast the prognosis OV patients.

Except malignant transformed cells, tumors consisted of normal cells, like fibroblasts, muscle cells, and inflammatory immune cells, which make up with TME together [14]. The interaction between tumor cells and TME influences the treatment effect of cancer [15]. In the early stage, tumors are infiltrated by various adaptive and innate immune cells, which conduct tumor-promoting and antitumor functions [16]. For example, higher infiltration level of CD8 T cells is usually related to better prognosis [17], while the macrophages M2 is supposed to be a poor prognostic marker [18]. In fact, immunotherapy has become one of the most hopeful methods in oncology. Clarifying the status of infiltrating immune cells in TME and understanding their number and function may contribute to formulate strategies to enhance the response rate of immunotherapy. Necroptosis is becoming a new target of cancer immunotherapy. Necroptosis in tumor cells regulates TME and antitumor immunity, which will be particularly helpful to the treatment of immune desert tumors [19, 20]. Necroptosis has different influence on tumor progression in the light of tumor cell types and TME [21]. However, the mechanisms are still unclear [22].

Redox reaction is a part of normal cell metabolism. If the redox homeostasis is damaged, cell death may be induced [23]. Increasing oxidative stress by increasing reactive oxygen species level or decreasing cell antioxidant capacity is a promising anticancer way, and it takes part in the mechanism of many chemotherapy drugs that have been used in clinical application [24]. More and more evidence shows that the redox modification participated in the regulation of some cell death modes, like necrosis and apoptosis. In addition, thiol redox switches involve in regulating the crosstalk between apoptotic and necrotic forms of cell death [25]. Mitochondrial peroxidase has a upregulated expression in different tumor types, including OV [26].

Our study calculated the expression profile of necroptosis-related genes (NRGs) and downloaded the immune pattern in OV by using two computational algorithms. In terms of NRG expression level, OV patients were separated into two independent subgroups. Then, the patients were divided into three gene clusters according to the differentially expressed genes (DEGs) between NRGclusters. A prediction signature was further built to predict the prognosis, so as to realize accurate identification and therapeutic measures of individuals.

## 2. Materials and Methods

### 2.1. Data Acquisition

Genetic and clinical profiles of OV were downloaded from the cancer genome atlas (TCGA) and gene expression omnibus (GEO). This study used 836 samples from two cohorts, TCGA-OV and GSE9891. Table S1 shows clinical features of individuals involved in the research. We combine TCGA-OV and GSE9891 datasets and use the “ComBat” algorithm to correct the batch effect. In order to reduce statistical bias, samples without overall survival (OS) value and without follow-up data were screened out from the study. OV patients with relevant characteristics (age, grade, and stage) and survival data were used for further analysis. GSE9891 was employed as an external set to validate.

### 2.2. Consensus Clustering Analysis

67 NRGs were obtained from published articles [27]. In accordance with gene expression data, we performed consumes clustering via R packages “ConsumusClusterPlus” [28]. To calculate the differences of NRGs in pathways, the gene set variation analysis (GSVA) was executed through a marker gene set (C2. Cp.kegg. V7.2) from Molecular Signatures Database.

### 2.3. Gene Clusters Identification on the Basis of DEGs

Firstly, the “limma” package was conducted to screen DEGs between gene clusters. The univariate Cox regression analysis was conducted on DEGs to screen DEGs related to OV survival. Secondly, OV patients were classified according to DEGs by using consistent clustering algorithm, and the patients were separated into three different subgroups.

### 2.4. Build Prognostic NRG Score Related to Necroptosis

After integrating the transcriptome and clinical data, we deleted individuals without prognostic data. All volunteers were randomly separated into training (*n* = 319) and testing subtypes (*n* = 318), and then the information of the training set was used to build the NRG score which related to OV patients. Based on prognostic genes associated with necroptosis, the least absolute shrinkage and selection operator (LASSO) regression algorithm was utilized to avoid over fitting through the “glmnet” R package. The multivariate Cox analysis identified key genes to establish predict model on the base of the training set. The formula is as follows: risk score = *Σ* (coefi × expi), where coefi and expi, respectively, mean the coefficient and express level of each gene. In accordance with median risk score, samples were divided into two different subgroups.

### 2.5. The Difference of Clinical Features Patients and Stratified Analysis

The score of individuals with different clinical features was compared by box diagram and scatter diagram. Hierarchical analysis was employed to assess the differences in OS between different subgroups using the Kaplan–Meier curve achieved by the “survminer” R package, to determine that the model still has the ability to predict under different clinicopathological characteristics.

### 2.6. Enrichment Analysis

Gene Ontology (GO) and Kyoto Encyclopedia of Genes and Genomes (KEGG) were applied to enrich NRG-related processes. KEGG is a dataset usually used to discover significantly altered pathways enriched in gene list. GO and KEGG were carried out through the bioinformatics platform [29]. By aggregating gene into gene sets, the gene set enrichment analysis (GSEA) offer rich scores, which allows users to have an in-depth understanding of how biological processes are influenced [30].

### 2.7. Assessment of Immune Infiltration Level

TME is widely involved in the tumorigenesis and tumor progression. ESTIMATE can predict the TME status in the light of relevant biomarkers expression in immune and stromal cells which conducted by the R package “estimate” [31, 32]. The single-sample gene set enrichment analysis (ssGSEA) can quantitatively estimate immune cell components from complex gene expression data by using the R package “GSVA” [33, 34]. CIBERSORT can quantify the abundance of TIICs in risk groups (http://cibersort.stanford.edu/).

### 2.8. The Difference of Biological Processes between NRGclusters

Rosenberg et al. defined a set of genes related to specific biological pathways, such as epithelial mesenchymal transition markers, DNA damage repair, and CD8 T-effector signature [35].

### 2.9. Phenotypes of RNAss Differentiation

In cancer stem cells (CSCs), mRNA expression-based RNAss is a variable to describe the similarity between tumors and stem cells [36, 37]. The higher the score was, the stronger the degree of stemness and the lower differentiation degree. RNAss scores were achieved from the xena (https://xenabrowser.net/datapages/).

### 2.10. Predict the Effect of Immunotherapy

Immunophenoscore (IPS) has been verified to predict patient response to immunotherapy [38], which can be achieved from The Cancer Immune Atlas (TCIA) (https://tcia.at/home). TMB can screen individuals who could benefit more from immunotherapy [39]. The burden of gain or loss of copy number variations (CNV) was calculated by gene pattern (https://cloud.genepattern.org) [40].

### 2.11. Analysis of Drug Sensitivity

To assess the therapeutic efficacy of chemotherapeutics on OV patients, the half maximum inhibitory concentration (IC50) of chemotherapeutics was achieved by the “prrophetic” R package [38]. We achieved the data of gene expression and drug sensitivity from CellMiner to calculate the correlation between some commonly used drugs and 8 genes.

### 2.12. Set Up a Nomograph

A nomogram can evaluate the OS through the “rms” package [41], where each factors were given a score, then added up them, and achieved a final score [42].

Hosmer-Lemeshow was applied to testify whether the predicted results were consistent with the fact [42]. The predictive ability of the model was explored through the *C*-index and area under curve (AUC) [43, 44]. *C*-index can be calculated by restricted mean survival (RMS). The capacity of nomogram was calculated by *C*-index and AUC, ranging from 0.5 to 1.0 [45].

### 2.13. Statistical Analysis

R version 4.1.0 was applied to analyses in this research. *P* < 0.05 was considered significant.

## 3. Results

### 3.1. Genetic and Transcriptional Changes of NRGs in OV

**Figure 1:**
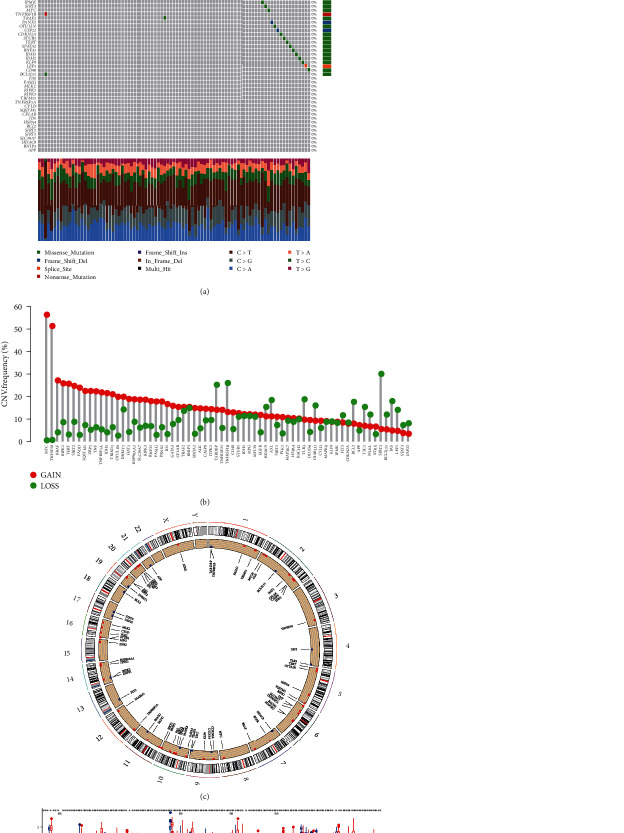
Heredity and transcriptional changes of necroptosis-related genes (NRGs) in OV. (a) In TCGA-OV population, 89 patients had gene mutations. (b) CNV frequency happened in NRG. (c) Location of CNV changes on 23 chromosomes in NRG. (d) Gene expression of NRGs in normal and tumor tissues. ^∗^*P* < 0.05, ^∗∗^*P* < 0.01, ^∗∗∗^*P* < 0.001.

The analysis flow chart is performed in Figure S1.  Figure 1(a) presents the summary outcome of the incidence of somatic mutations in 67NRGs.There were 89 mutations that occurred in 436 samples (20.41%) where ATRX and ALK had highest mutation frequency (2%). The CNV of MYC and TNFSF10 increased significantly, while the CNV of TARDBP, TNFRSF21, HDAC9, AXL, TLR3, and CYLD were decreased (Figure 1(b)). The location of CNV of 67NRGs on chromosome is exhibited in Figure 1(c). We also compared the discrepancy of genes in control and OV samples (Figure 1(d)). Among the 67 genes, 38 genes had different expression levels, and the corresponding OS was also different (Figure S2). Genetic and transcriptional levels of NRGs between OV and control tissues were different which means that NRGs have a significant role in the progression of OV.

### 3.2. Discrimination of NRGclusters in OV

**Figure 2:**
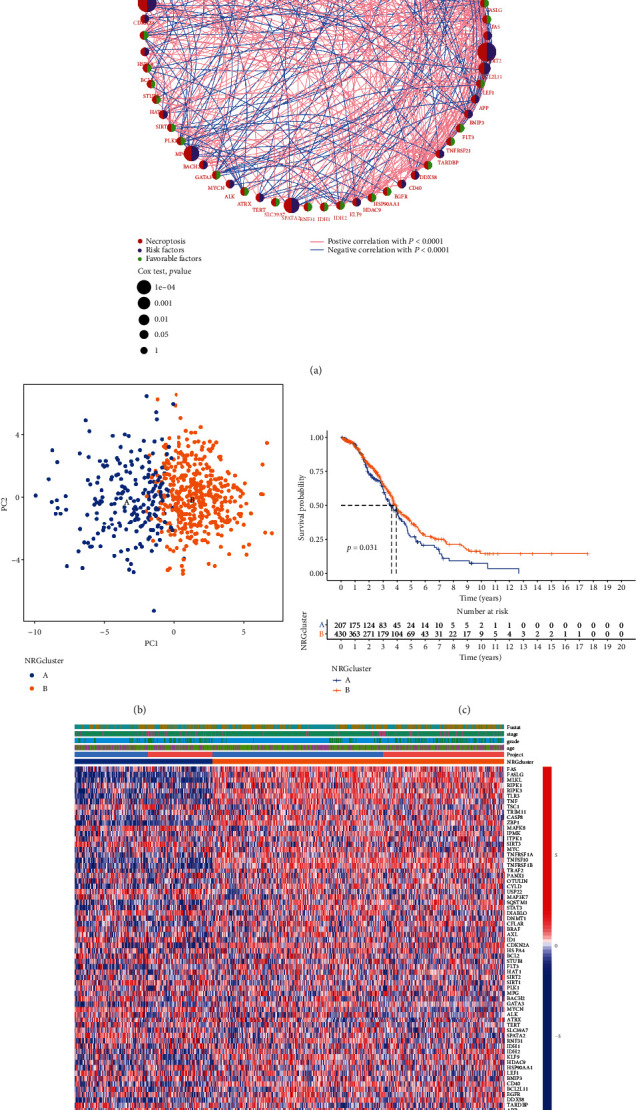
Clinical and biological factors of two clusters defined by clustering analysis. (a) Interrelationships among NRGs in OV. (b) PCA scatter plot reflecting the distinction between NRGclusters. (c) Survival probability of NRGcluster A and NRGcluster B. (d) Difference of clinical factors expression levels of NRGs between NRGclusters.

The state of NRG interactions and regulator connections OV populations are presented in Figure 2(a). For further analysis of the express characteristics of NRG in OV, we classified OV patients from *k* = 1 to *k* = 9 (Figure S3). PCA showed discrepancies in necroptosis transcription between the NRGclusters (Figure 2(b)). The Kaplan–Meier curve implied that NRGcluster A had higher survival probability than patients in NRGcluster B (Figure 2(c)). Furthermore, there were discrepancies in NRG expression and clinicopathological features among different OV subtypes (Figure 2(d)). Compared with NRGcluster B, patients in NRGcluster A had older age, more advanced stage and grade, and worse survival status.

### 3.3. Characteristics of Different Subtypes of TME

**Figure 3:**
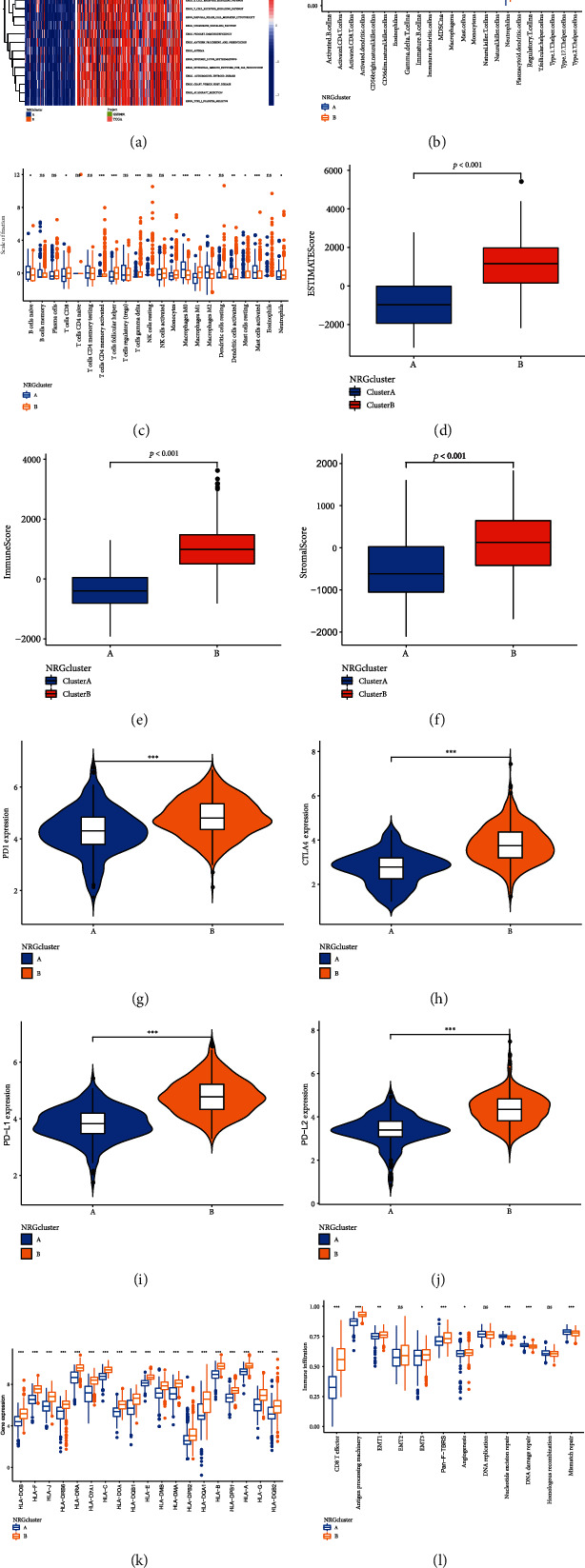
Tumor mutation load of the two NRGclusters. (a) Gene set enrichment analysis of NRGclusters. (b, c) Immune infiltration levels of two NRGclusters. (d) The stromal score, (e) immune score, and (f) estimated score of the two NRGclusters were compared. (g–j) Immune checkpoint expression of NRGclusters. (k) HLA expression of two NRGclusters. (l) Compare the scores of biological pathways between the two NRGclusters. ^∗^*P* < 0.05, ^∗∗^*P* < 0.01, ^∗∗∗^*P* < 0.001.

As performed in Figure 3(a), some immune activation-related processes like B cell receptor signaling pathway were enriched in NRGcluster B, which indicate immune activation (Figure 3(a)). The immune infiltration scores of NRGcluster A and NRGcluster B were compared, showing a great difference. The immune infiltration level in NRGcluster A was lower than that in NRGcluster B. Innate and adaptive immune cells were enriched in NRGcluster B (Figure 3(b)). Then, we calculated the association between two RNA modified subtypes and 22 TIICs. The proportion of immune cells was higher in NRGcluster B which means that NRGcluster B may related to immune activation (Figure 3(c)). Therefore, we estimated the TME score of the NRGclusters (Figures 3(d)–3(f)). A higher estimate score represents a higher fraction of stromal and immune cells. The outcomes indicated that patients in NRGcluster B had a higher TME score. We noticed that the two NRGclusters had different immune infiltrations. Features of NRGcluster A were similar to the definition of “cold” tumors, which has less invasive immune cells and may achieve less benefit from immune therapy, while NRGcluster B is roughly similar to “hot” tumors. Regarding the express levels of immune checkpoints in two NRGclusters, we noticed that PD1 (Figure 3(g)), CTLA4 (Figure 3(h)), PDL1 (Figure 3(i)), and PD-L2 (Figure 3(j)) had a high expression level in NRGcluster B. The HLA expression of the NRGclusters was also different (Figure 3(k)). We then found that some immune-related pathways were more prominent in NRGcluster B (Figure 3(l)).

### 3.4. Identification of Gene Cluster Based on DEGs

**Figure 4:**
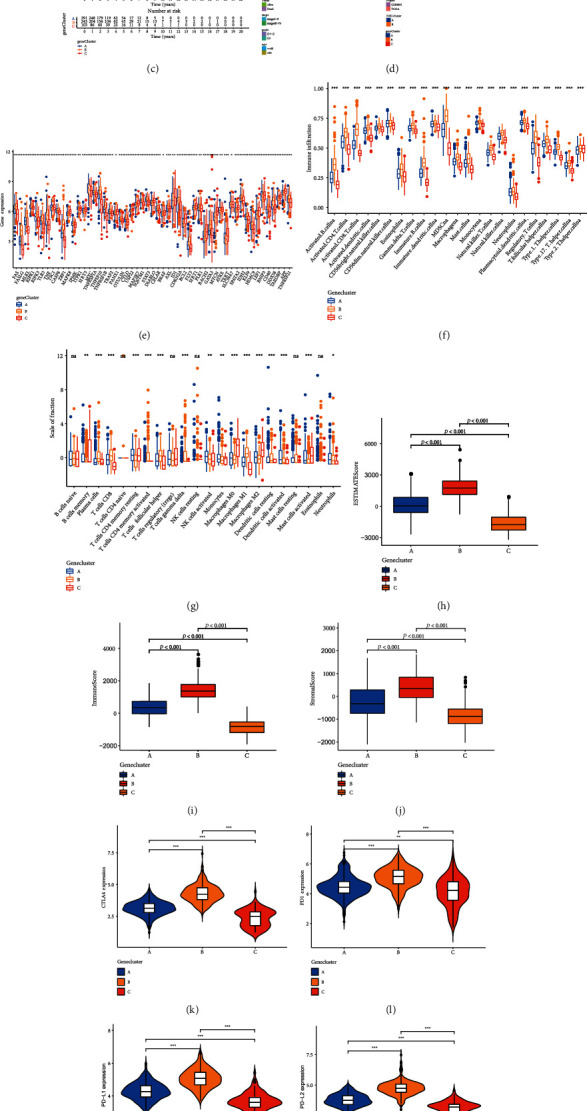
Identify gene subtypes in the light of DEGs (a) GO and (b) KEGG enrichment analysis of two gene clusters. (c) Survival probability of gene subtypes. (d) Clinicopathological features of three gene subtypes. (e) The expression of NRGs of gene clusters. (f, g) Immune infiltration of gene clusters. (h) Stromal score, (i) immune score, and (j) estimated score of three gene clusters were compared. (k–n) Expression of immune checkpoints. (o) HLA expression level of gene clusters. (p) Compare the score of biological pathways between the three gene clusters. ^∗^*P* < 0.05, ^∗∗^*P* < 0.01, ^∗∗∗^*P* < 0.001.

We screened out DEGs (Figure S4a) and then conducted GO and KEGG analyses which revealed that NRGs were mainly associated with immune-related processes which means that they are crucial in the immune regulation of TME (Figures 4(a) and 4(b)). The best number of clusters is three (Figure S4b-S4e). Gene cluster B had the highest survival probability and was related to early stage, early grade, younger age, and better survival status (Figures 4(c) and 4(d)). In addition, patients in gene cluster B and NRGcluster B had similar clinical characteristics. The gene expression in three gene clusters was different (Figure 4(e)). The outcome of ssGSEA showed that the vast immune cells had higher infiltration levels in gene cluster B (Figure 4(f)), which was primarily infiltrated by adaptive immune cells (Figure 4(g)). In addition, gene cluster B tends to have higher TME score and immune checkpoints expression (Figures 4(h)–4(n)). HLA expression levels of gene clusters in the three groups were also the highest expression in gene cluster B (Figure 4(o)). In addition, classical biological progresses were more prominent in gene cluster B (Figure 4(p)). According to these features, we considered that gene cluster B belongs to “hot” tumors.

### 3.5. Build and Testing the Prognostic Signature

**Figure 5:**
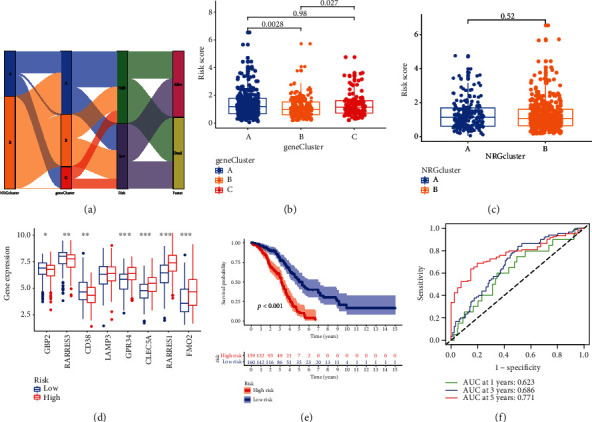
Construct NRG score based on the training set. (a) Distribution of groups with different classification criteria. Difference of NRG score between (b) gene clusters and (c) NRGclusters. (d) Expression of the 8 NRGs between risk groups. (e) The Kaplan–Meier analysis shows survival probability. (f) Assess sensitivity and specificity NRG score prediction in 1, 3, and 5 years. ^∗^*P* < 0.05, ^∗∗^*P* < 0.01, ^∗∗∗^*P* < 0.001.

Figure 5(a) displays the spread of patients in three gene clusters and two NRGclusters. 14 OS-related genes were screened as candidate prediction genes (Figure S5a-S5b). Finally 8 achieved genes (GBP2, RARRES3, CD38, LAMP3, GPR34, CLEC5A, RARRES1, and FMO2) were selected. Among them, GBP2, RARRES3, and CD38 were protective genes (Figure S5c). According to the above results, we assessed the NRG score: risk score = (−0.5265 × GBP2 expression) + (−0.2565 × RARRES3 expression) + (−0.2786 × CD38 expression) + (0.2053 × LAMP3 expression) + (0.4263 × GPR34 expression) + (0.2201 × CLEC5A expression) + (0.2249 × RARRES1 expression) + (0.1025 × FMO2 expression). We noticed that the score of gene cluster B was lower than that of gene cluster A (Figure 5(b)). In the grouping according to NRGcluster, there was no diversity of risk score between the two subgroups (Figure 5(c)). Most of the 8NRGs had different expression between the two risk subgroups in training set (Figure 5(d)). Individuals with low scores had higher OS when compared with higher score group (Figure 5(e)). In addition, the AUC of NRG score for 1, 3, and 5 years were 0.623, 0.686, and 0.771, respectively (Figure 5(f)). Then, we use the testing group, all groups from TCGA, and the data from GEO to verify the above results. Figure S6 presents the difference of NRGs expression, survival analysis, and AUC in two risk groups in testing set, all sets, and GEO set, respectively. There were distinctions in the eight gene expressions between the risk groups. The AUC of NRG score for 1, 3, and 5 years is still high, which means that the model had excellent predict ability.

### 3.6. The Difference of Risk Score between Different Feature Patients

To analyze the relationship between NRG score and clinical features, we compared the risk scores of different individuals. It was found that the NRG score of OV patients in stages I-II was lower than in stages III-IV (Figure S7a). Moreover, the risk score of OV patients with better survival status was also lower than that of OV patients with worse survival status (Figure S7b). The NRG score was proved to be an independent prognostic variable (Table 1). In different age, stage, and grade subgroups, the OS of the high NRG score group tends to be lower (Figure S7c). Furthermore, for BRCA1 wild patients and chemotherapy treated patients, the survival probability of patients with higher risk score was lower.

### 3.7. Assessment of TME in Terms of the NRGs

**Figure 6:**
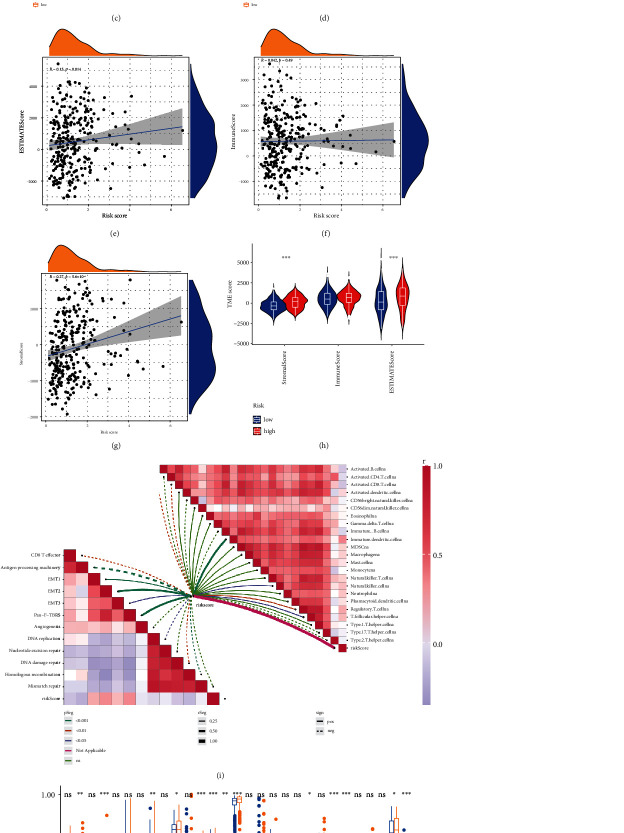
Evaluate TME of different risk groups. Main enriched biological pathways of (a) low NRG score group and (b) high-score group. (c, d) The ssGSEA score and immune infiltration score of risk groups. (e–h) The NRG score had positive association with stromal cells, immune cells, and estimated score. (i) The relevance of risk score and immune cells as well as classical biological pathway score. (c, j) The difference of immune function score betweenthe groups.

**Figure 7:**
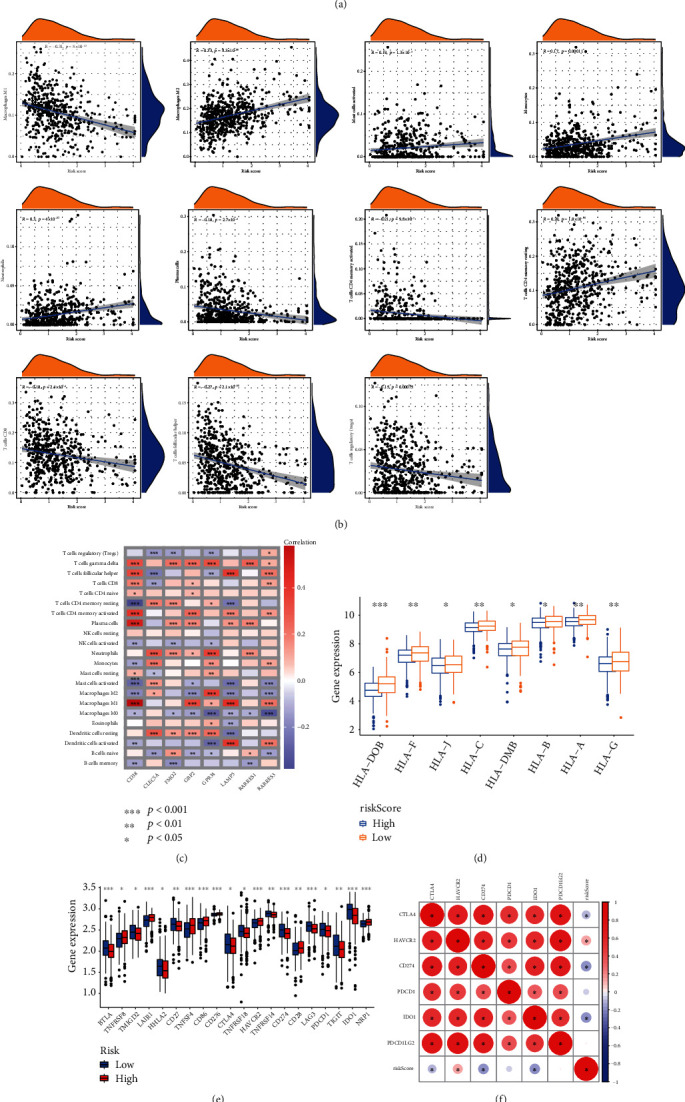
Immune infiltration situations of the subtypes. (a) The difference of immune cell abundance between the groups. (b) The correlation between 8 genes and immune cell abundance. (c) The relationship between risk score and immune cell abundance. (d) The difference of HLA expression between the groups. (e) Twenty immune checkpoints with differential expression in the two groups were depicted. (f) The correlation between immune checkpoints and risk score. (g) The differences of IPS cell expression. ^∗^*P* < 0.05, ^∗∗^*P* < 0.01, ^∗∗∗^*P* < 0.001.

For the purpose of deepen understanding the TME of subgroups, we first conducted GSEA and found that the low NRG score group was mainly related with some immune-related processes (Figure 6(a)), while high-score individuals were associated with cancer-related processes (Figure 6(b)). Further studies indicated that pan-F-TBRS was obviously activated in the high group (Figure 6(c)). People with higher risk score had higher immune score and stromal score (Figures 6(e)–6(g)). Not surprisingly, the combined estimated score of these two scores was also higher in the high-risk group. We then identified the relationships between immune cells and risk score as well as the correlations between risk score and the score of classical biological pathways enrichment (Figures 6(h) and 6(i)). The immune function score of B cells, T helper cells, and Tfh was significantly higher in low-risk groups (Figure 6(j)). Then, we calculated the correlation between risk score and immune cell abundance (Figure 7(a)). The NRG score was positively associated with macrophages M2, mast cells activated, monocytes, neutrophils, and T cells CD4 memory resting and had negative relationship with macrophages M1, plasma cells, etc. (Figure 7(b)). In terms of oxidative stress, the expression of oxidative stress-related genes was low in low-risk group, especially CYBB (Figure S8). Moreover, we noticed that a great deal of immune cells was related to the genes (Figure 7(c)). Human leukocyte antigen expression was also higher in lower risk cohorts (Figure 7(d)).  Figure 7(e) shows that many immune checkpoints were overexpressed in patients in high risk. There was discrepancy of immune checkpoints expression between the groups. CTLA4, CD274, PDCD1, and IDO1 had negatively correlation with risk score, and HAVCR2 has positive relationship with the score (Figure 7(f)). The outcome of IPS score indicates that low-risk score was associated with higher immunogenicity (Figure 7(g)).

### 3.8. Predict the Curative Effect of Immunotherapy

**Figure 8:**
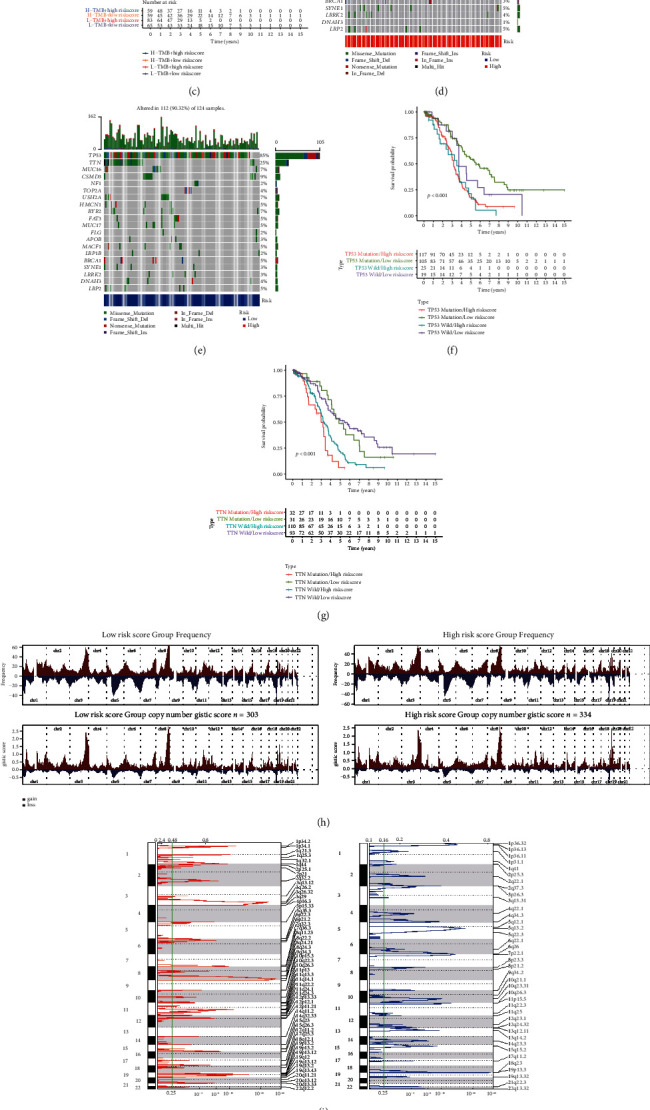
Assess the efficacy of immunotherapy. (a) RNAss was negatively correlated with NRG score. (b, c) Survival probability of people with different TMB and risk score. (d, e) The situation of gene mutation in different risk groups. (f, g) Survival probability of people with TP53 mutation and TTN mutation. (h) Copy number score for the groups. (i, j) Cytoband shows amplification (left) and deletion (right).

Figure 8(a) shows the relationships between the NRG score and CSC index. We noticed that the NRG score was negatively associated with CSC index (*r* = −0.35, *P* < 0.001) which showed that CRC cells with lower risk score have more obvious stem cells and lower cell differentiation. L-TMB accompanied with high risk means a significant poorer survival probability than other groups (Figures 8(b) and 8(c)). Mutation frequencies of TP53 and TTN were high in both cohorts (Figures 8(d) and 8(e)). Accompanied with high-risk score, OS becomes lower (Figures 8(f) and 8(g)).  Figure 8(h) shows the distribution of GISTIC scores on all chromosomes. Focal amplification and deletion of different chromosome regions were found (Figures 8(i) and 8(j)).

### 3.9. Estimation of Drug Sensitivity

We chose drugs usually applied in the remedy of OV to assess the sensitivity of patients to these drugs. IC50 values of docetaxel in high-risk patients were lower, while IC50 of A.443654 and paclitaxel was lower in people with low-risk score (Figure S8a-S8c). We also calculated the relationships between some common drugs and 8 genes. Taken together, the above results suggested that NRGs are correlated with drug sensitivity (Figure S8d).

### 3.10. Construct Nomograms for Clinical Application

**Figure 9:**
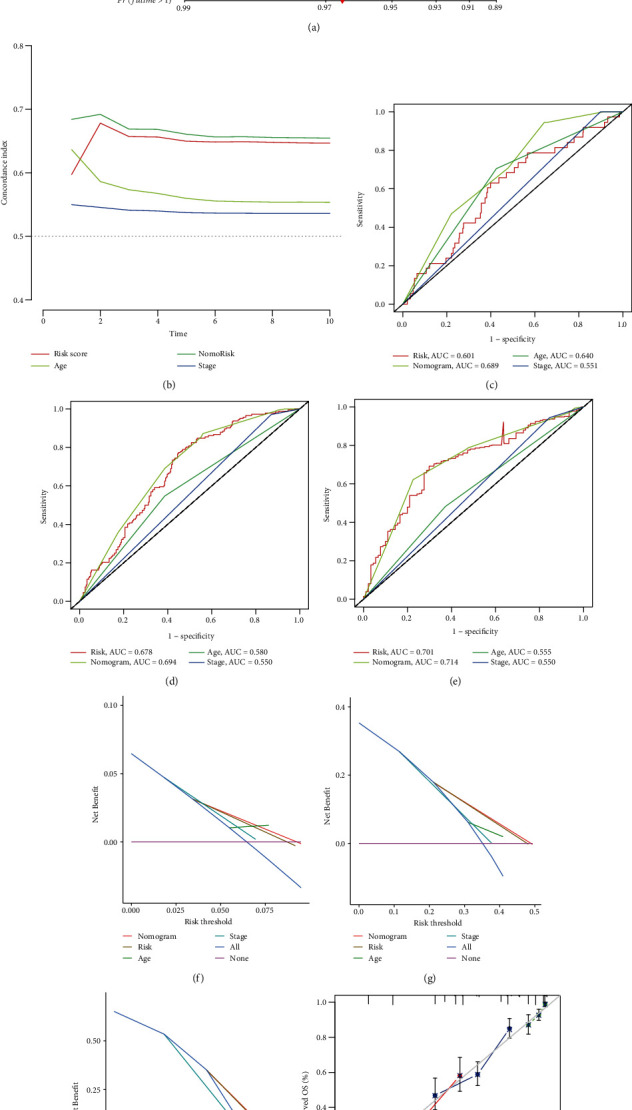
Built and verification of nomogram. (a) Nomograms used to predict OS in 1, 3, and 5 years. (b) *C*-index of prognostic factors including risk score. (c–h) ROC and DCA curves of 1, 3, and 5 years. (i) Nomogram calibration curve of the model.

Taking care of the practical utilize of NRG score in predicting, we built a nomogram containing NRG score and clinical factors (Figure 9(a)), predictors including NRG risk score, age, nomogram risk, and stage. Our signature had higher *C*-index (Figure 9(b)). The AUC corresponding to NRG risk was generally higher which indicates great prediction performance, and it will be better when considering age and stage (Figures 9(c)–9(e)). DCA indicated that the NRG risk score or nomogram risk combined with clinical features had a higher benefit in predicting the OS of OV patients at 1, 3, and 5 years (Figures 9(f)–9(h)). A subsequent calibration diagram proved it again (Figure 9(i)).

### 3.11. NRG Model Has Great Prognostic Performance

To contrast the prognosis ability of our signature with other signatures, we screened four prognosis models from the previous literatures. We used the multivariate Cox regression analysis to assess the estimate score, based on specific genes expression (Figure S10a). Figure S10b indicated that the prognosis of high-risk individuals was worse in all four models. Obviously, our model has the highest *C*-index which was 0.65 (Figure S10c). Therefore, our genetic characteristics performed best in about six years (Figure S10d).

## 4. Discussion

Despite progress in study and remedy of OV, the 5-year survival rate is still low [46], and more than half of the patient relapse and develop drug resistance [47–50]. Cell death inhibition is the ultimate cause of drug resistance in OV [51]. As the main type of cell death, previous studies mostly focused on the drug resistance of apoptosis in OV [52]. Necroptosis is a newly noticed type of regulatory necrosis which has been proven to have great effect on cancer, especially in drug resistance [53]. Therefore, our exploration may improve the poorer outcome of OV.

Patients in NRGcluster A had more advanced clinical characteristics and poorer survival than patients in NRGcluster B. There are also distinctions in the features of TME between the two NRGclusters. The TME score and immune checkpoint expression was higher in NRGcluster B patients. We screened three gene clusters in the light of DEGs. Then, we established an effective prognostic risk score and verified its predict performance. There were great differences in clinical features, TMB, TME, immune checkpoint, *C*-index, CNV, and drug sensitivity between the risk groups. Finally, the nomogram was established to further enhance the performance and promote use of NRG score.

The prediction model has a close correlation with redox stress and immune environment. The high-risk group has higher levels of redox stress, which may be closely related to their poor prognosis. Despite advances has been achieved in immunotherapy recently, outcomes of OV patients have still been heterogeneous, indicating the effect of TME in the occurrence and development of OV [54].TME is an ecosystem consisting of tumor cells, infiltrating immune cells and stromal cells intertwined with noncellular components. In this study, the necroptosis pattern with immune inhibition was related to higher NRG score, and the necroptosis pattern with immune activation was related to lower NRG score. Macrophages M1, also known as “classic activated macrophages,” has a proinflammatory effect. Their high expression was associated with a better prognosis in patients with OV [55]. OV-associated memory T cells are also associated with chemotherapy response and longer survival [56]. CD4 T cells have crucial effect on almost every aspect of immunity and are considered an important component needed in tumor immunotherapy [57]. Plasma cell infiltration is related with high CD4 and CD8 T cell response and great prognosis [58, 59], while NRG score was positively associated with macrophage M2, mast cell activation, monocyte, neutrophil, and T cell CD4 memory rest. The more macrophages M2, the worse the prognosis of patients with advanced OV [60]. Tumors with high mast cell are associated with immunosuppressive OV TME and are potentially insensitive to immunotherapy [61]. Monocytes are recruited around the cancer and differentiated into macrophages, which can be used as biomarkers of OV progression [62]. Neutrophils are key players in OV and have been considered new biomarkers of cancers or as immunotherapy targets to promote tumor progression [14].

TME is closely correlated with the response of patients with various cancers to immunotherapy, and patients with immunodominant TME subtypes benefit the most from immunotherapy [63]. In the past decades, immunotherapy, especially the treatment using ICIs, was developing rapidly. The researches on ICI are booming, and clinical studies have proved their safety and effectiveness. In this study, we observed that CTLA4, CD274, PDCD1, and IDO1 had a negative correlation with risk score, and HAVCR2 has a positive association with risk score. Among them, researchers have a deeper understanding of CTLA4 and PDCD1. Evidence from non-OV shows that patients with hot tumors infiltrated by immunogenic T cells have lasting clinical benefits in PD-1/PD-L1 blocking response compared with individuals with cold tumors [64]. However, their effect in OV is not clear. Whether they can be used as targets of immunotherapy in clinical still needs further research. Immunotherapy conducted on these patients may obtain better curative effect.

In addition, we found that OS decreased significantly when TP53 and TTN mutation particularly combined with high-risk score. Proteins encoded by some major target genes regulated by TP53 are essential for maintaining genomic integrity and cell life cycle [65]. TTN mutations are closely associated with the response to immune checkpoint blockade (ICB) [66, 67]. However, previous articles have not clearly discussed whether TTN or TP53 mutation has an impact on the immunotherapy effect of OV. Human CNV is a repetitive or missing DNA fragment relative to the reference genome, which may lead to genomic imbalance and diseases such as tumor. So, it is correlated with the process of diagnosis and prognosis [68, 69]. CNV has been tested to be related to the prognosis of OV [70]. Low-risk patients have more gene mutations, and CNV load belongs to immune activation subgroup.

However, immunotherapy using ICB alone is less effective in the treatment of OV [71]. Therefore, it is necessary to treat OV patients by ICB combined with chemotherapy, radiotherapy, and other therapeutic methods. Cisplatin and its derivatives are commonly used in OV chemotherapy. It has been determined that cisplatin can induce necroptosis and significantly increase the death of OV cells, to improve the anticancer effect of chemotherapeutics [72, 73]. Taxane cytotoxic drugs such as docetaxel have become one of the most effective drugs for the immunotherapy of gynecological cancer recently. It has been approved for the remedy of OV, breast cancer, and so on [74, 75]. Paclitaxel can induce immunogenic cell death in OV and achieve therapeutic effect [76]. We found IC50 values of docetaxel in high-risk patients were lower, while IC50 values of A.443654 and paclitaxel were lower in low-risk patients. Therefore, different chemotherapy drugs can be used for patients in different risk groups, which may get better therapeutic effect.

This study also has some shortcomings. Firstly, the necroptosis genes included in this study were achieved from previous articles. Some unreported NRGs might be ignored. Secondly, the prognostic model constructed for OV in this study needs to be verified in clinical application. Therefore, we need to screen new genes related to necroptosis in more cohorts and collect enough cases and clinical information of OV in the future, to ensure that the model is effective for clinical application.

## 5. Conclusions

Our comprehensive analysis of NRG reveals its impact on TME, clinical characteristics, and prognosis of OV. The therapy role of NRGs in immunotherapy was also analyzed. The above results emphasize the significance of NRGs and offer new orientation for guiding precision therapy strategy of OV patients.

## Figures and Tables

**Table 1:** Univariate and multivariate Cox regression analyses of the prognosis-related variables.

Variable	Univariable model				Multivariable model			
	HR	HR.95 L	HR.95H	*P* value	HR	HR.95 L	HR.95H	*P* value
Training set								
Age	1.4679	1.0659	2.0215	0.0187	1.4287	1.0361	1.9702	0.0296
Grade	1.1163	0.7573	1.6454	0.5784				
Stage	11.9886	1.6752	85.7985	0.0134	9.0377	1.2600	64.8256	0.0285
Risk score	1.9319	1.6308	2.2885	0.0001	1.8825	1.5865	2.2338	0.0001
Testing set								
Age	1.4023	1.0347	1.9005	0.0293	1.3802	1.0179	1.8715	0.0380
Grade	1.2773	0.8671	1.8814	0.2156				
Stage	2.7702	1.2974	5.9149	0.0085	2.4765	1.1551	5.3098	0.0198
Risk score	1.3176	1.1442	1.5173	0.0001	1.2787	1.1047	1.4800	0.0010
All set								
Age	1.4182	1.1398	1.7645	0.0017	1.3772	1.1066	1.7139	0.0041
Grade	1.2049	0.9166	1.5839	0.1816				
Stage	3.9317	1.9467	7.9407	0.0001	3.3380	1.6495	6.7551	0.0008
Risk score	1.4904	1.3431	1.6538	0.0001	1.4498	1.3031	1.6130	0.0001
GEO set								
Age	1.5109	1.0347	2.2063	0.0326	1.5138	1.0358	2.2124	0.0322
Grade	1.3183	0.8887	1.9555	0.1696				
Stage	6.8898	2.1835	21.7396	0.0010	5.9038	1.8625	18.7139	0.0026
Risk score	1.3764	1.1772	1.6093	0.0001	1.3164	1.1175	1.5506	0.0010

## Data Availability

The data used to support the findings of this study are available from the corresponding author on reasonable request.

## Abbreviations

NRGs: Necroptosis-related genes
OV: Ovarian cancer
TME: Tumor microenvironment
LASSO: Least absolute shrinkage and selection operator
CNV: Copy number variations
TIL: Tumor infiltrating lymphocyte
DEG: Differentially expressed gene
TCGA: The cancer genome atlas
GEO: Gene Expression Omnibus
OS: Overall survival
PCA: Principal component analysis
GSVA: Gene set variation analysis
ROC: Receiver operating characteristic curve
GO: Gene Ontology
KEGG: Kyoto Encyclopedia of Genes and Genomes
GSEA: Gene set enrichment analysis
ssGSEA: Single-sample gene set enrichment analysis
CSC: Cancer stem cell
IPS: Induced pluripotent stem
TMB: Tumor mutation load
ICI: Immune checkpoint inhibitor
IC50: Half maximum inhibitory concentration
NCI-60: 60 types of cancer cell
NCI: National Cancer Institute's Center for Cancer Research
AUC: Area under curve
DCA: Decision curve analysis
RMS: Restricted mean survival
ICB: Immune checkpoint blockade
TIIC: Tumor immune infiltrating cells.

## Supplementary Materials

Supplementary Materials Figure S1: flow chart of this study. Figure S2: OS of patients with different level of NRGs. Figure S3: clustering analysis determine the best number of clusters. (a-b) Uniform clustering cumulative distribution function (CDF) and the change of area under CDF curve, *k* = 2 − 9 (*k* means the number of clusters). (c) The samples were divided into two clusters when *k* = 2. (d) Tracking plot of the cluster. Figure S4: clustering analysis determine the best number of clusters. (a-b) CDF and the area under CDF curve from k equal two to *k* equal 9. (c) The samples were divided into two clusters when *k* = 3. (d) Tracking plot of the cluster. Figure S5: identifying genes with predict potential. (a-b) LASSO regression analysis and partial likelihood deviance on the prognostic genes. (c) Forest plot shows the result of multivariate Cox regression analysis. Figure S6: validate prognostic ability of NRG model expression level of NRGs in testing set (a), all set (b), and GEO set (c). Kaplan–Meier survival corves of samples in testing set (d), all set (e), and GEO set (f). ROC curves of 1, 3, and 5 year survival in testing set (g), all set (h), and GEO set (i). Figure S7: attest the prognosis performance of the model by stratified analysis. Compare the risk scores of individuals in different stages (a) and had different fustat (b). (c) Comparing survival time of patients with different clinical features. Figure S8: expression of oxidative stress-related genes in the groups. ^∗^*P* < 0.05, ^∗∗^*P* < 0.01, ^∗∗∗^*P* < 0.001. Figure S9: evaluate drug sensitivity. The IC50 of A.443654 (a), docetaxel (b), and paclitaxel (c) in samples. (d) Correlativity of common drugs and gene expression level. Figure S10: compare NRG model with some established models (a-b) ROC curves and KM curves of four published gene-signatures. (c-d) *C*-index and RMS time curve of prognostic models.
