# Supplementary material for: Necroptosis-Related Modification Patterns Depict the Tumor Microenvironment, Redox Stress Landscape, and Prognosis of Ovarian Cancer
Source: Oxid Med Cell Longev. 2023 Apr 11;2023:4945288. doi: 10.1155/2023/4945288 (PMC10113055; doi:10.1155/2023/4945288)
Supplement: Supplementary Materials — Figure S1: flow chart of this study. Figure S2: OS of patients with different level of NRGs. Figure S3: clustering analysis determine the best number of clusters. (a-b) Uniform clustering cumulative distribution function (CDF) and the change of area under CDF curve, k = 2 − 9 (k means the number of clusters). (c) The samples were divided into two clusters when k = 2. (d) Tracking plot of the cluster. Figure S4: clustering analysis determine the best number of clusters. (a-b) CDF and the area under CDF curve from k equal two to k equal 9. (c) The samples were divided into two clusters when k = 3. (d) Tracking plot of the cluster. Figure S5: identifying genes with predict potential. (a-b) LASSO regression analysis and partial likelihood deviance on the prognostic genes. (c) Forest plot shows the result of multivariate Cox regression analysis. Figure S6: validate prognostic ability of NRG model expression level of NRGs in testing set (a), all set (b), and GEO set (c). Kaplan–Meier survival corves of samples in testing set (d), all set (e), and GEO set (f). ROC curves of 1, 3, and 5 year survival in testing set (g), all set (h), and GEO set (i). Figure S7: attest the prognosis performance of the model by stratified analysis. Compare the risk scores of individuals in different stages (a) and had different fustat (b). (c) Comparing survival time of patients with different clinical features. Figure S8: expression of oxidative stress-related genes in the groups. ∗P < 0.05, ∗∗P < 0.01, ∗∗∗P < 0.001. Figure S9: evaluate drug sensitivity. The IC50 of A.443654 (a), docetaxel (b), and paclitaxel (c) in samples. (d) Correlativity of common drugs and gene expression level. Figure S10: compare NRG model with some established models (a-b) ROC curves and KM curves of four published gene-signatures. (c-d) C-index and RMS time curve of prognostic models. [file 4945288.f1.pdf]

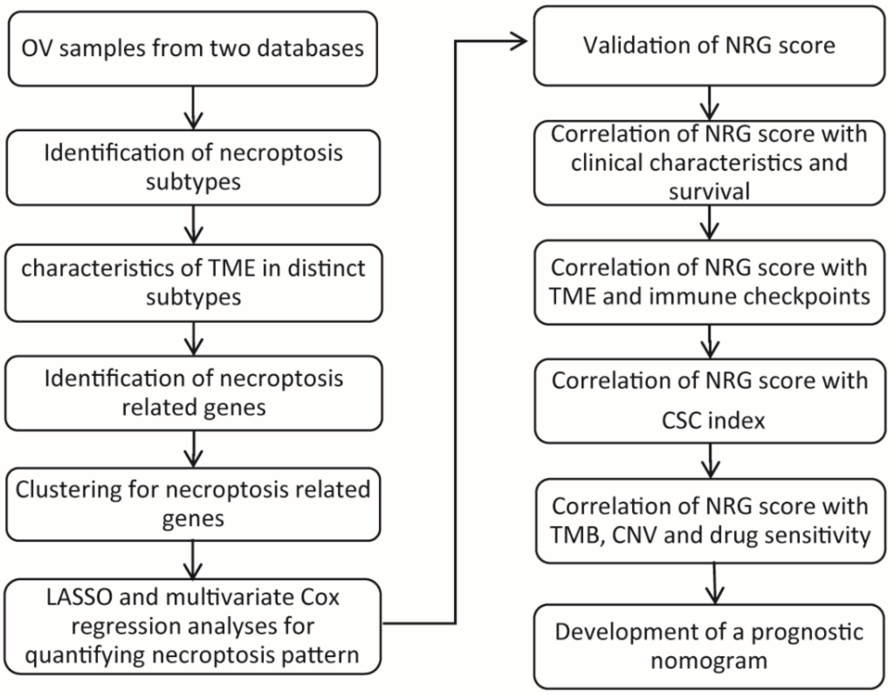

Fig S1 The analysis process of this study

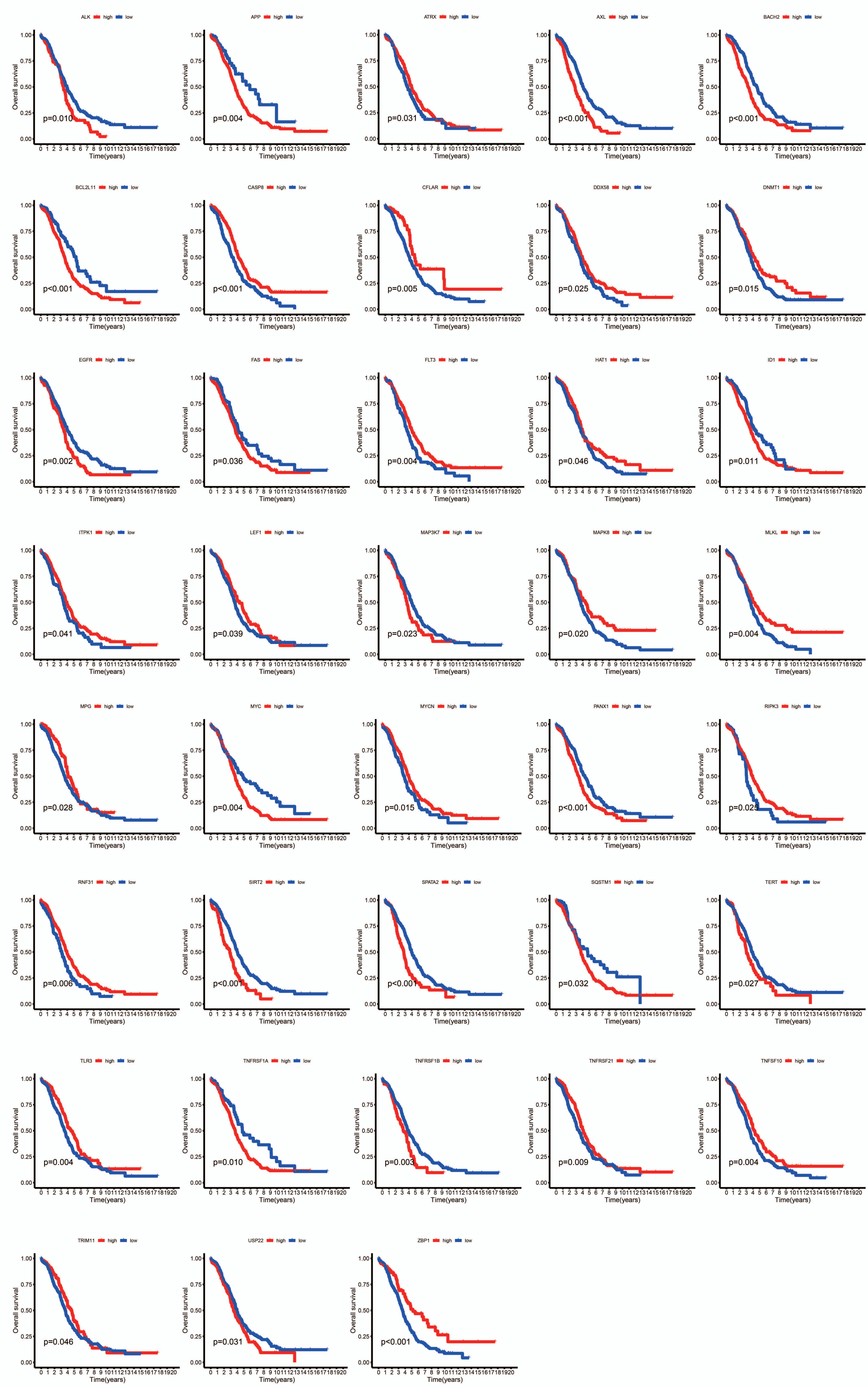

Fig S2 OS differences corresponding to different gene expression level.

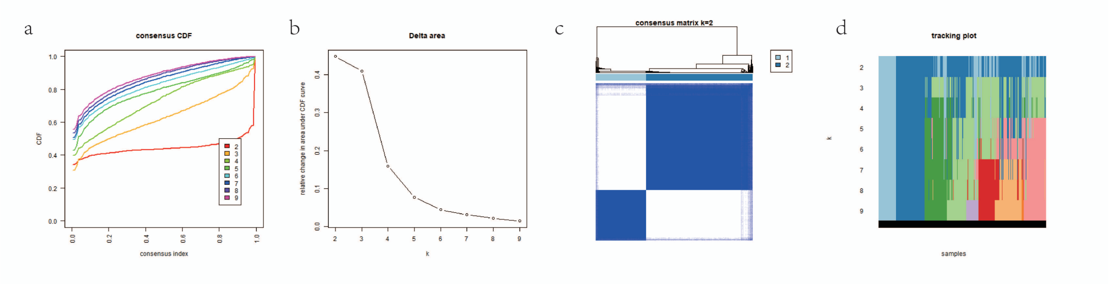

Fig S3 Consensus clustering analysis

(a) Uniform clustering cumulative distribution function (CDF), k = 2-9 (k represents the number of clusters). (b) The change of area under CDF curve with k = 2-9. (c) The samples were divided into two clusters when k=2. (d) Tracking plot of the cluster when k=2.

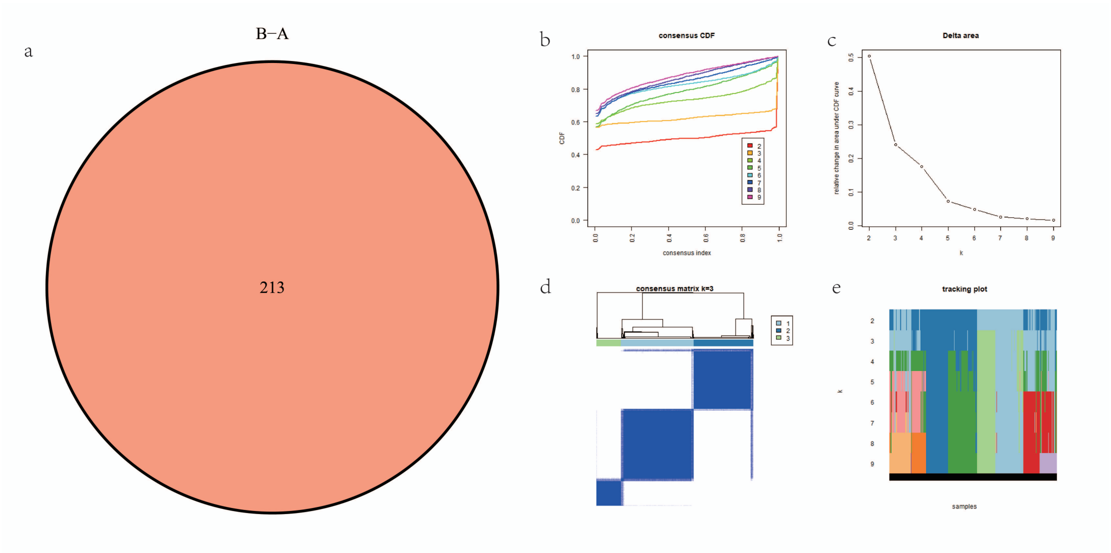

Fig S4 Consensus clustering analysis

(a) Venn diagram showing the similar parts between NRGclustr A and NRGcluster B. (b) Uniform clustering cumulative distribution function (CDF), k = 2-9 (k represents the number of clusters). (c) The change of area under CDF curve with k = 2-9. (d) The samples were divided into three clusters when k=3. (e) Tracking plot of the cluster when k=3.

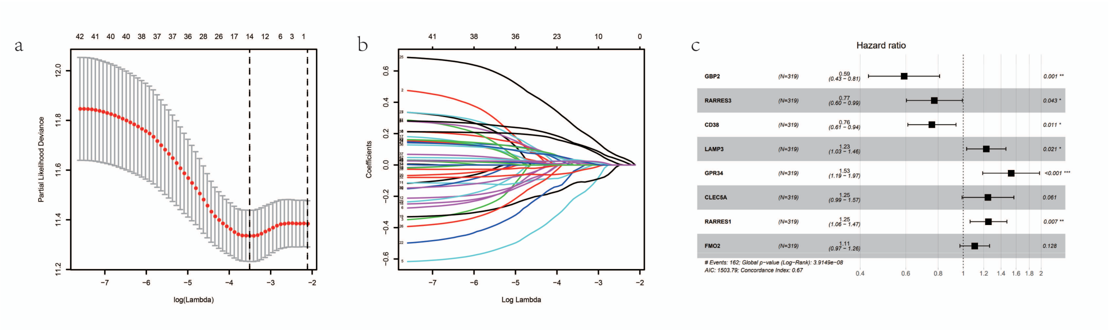

Fig S5 Identifying representative candidate prognostic genes

(a-b) The LASSO regression analysis and partial likelihood deviance on the prognostic genes. (c) Forest plot of multivariate cox regression analysis for prognostic genes.

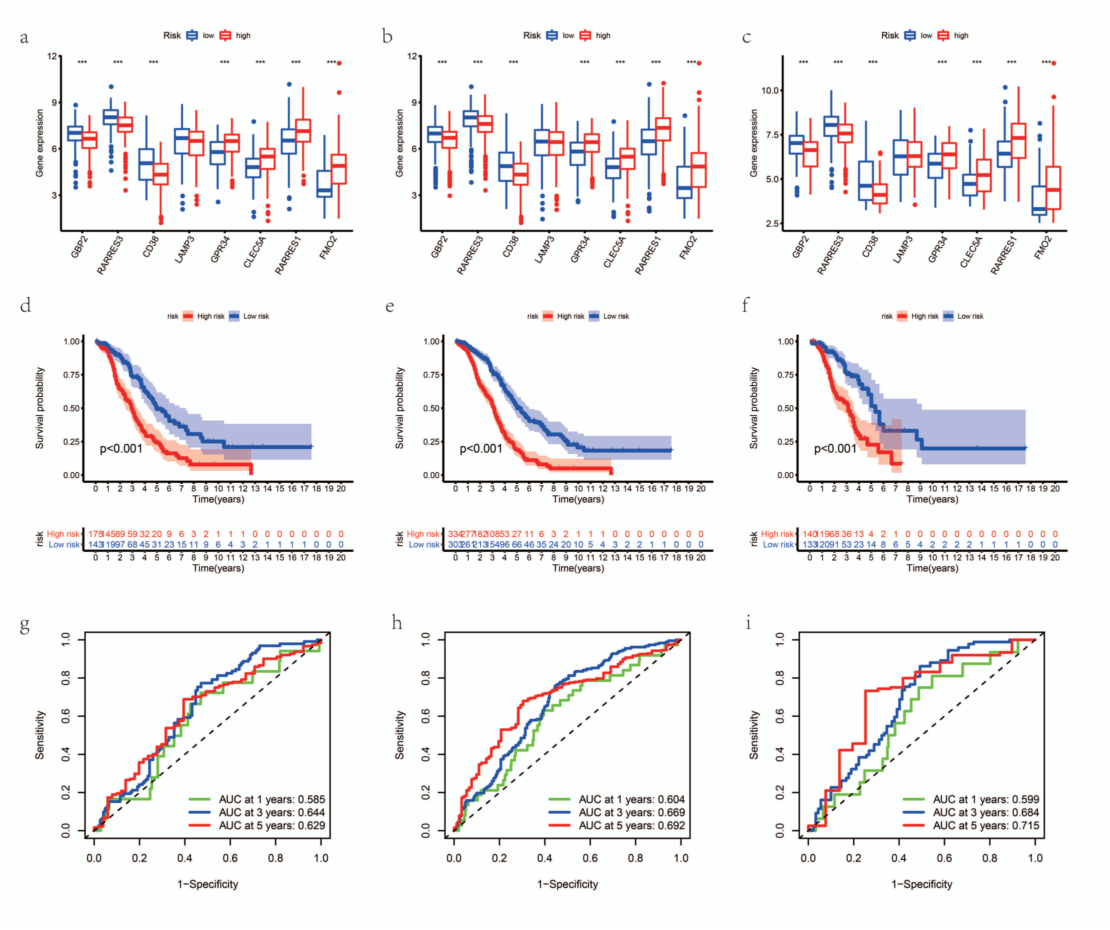

Fig S6 Validation of the prognostic model based on NRG score

Different expression of the 8 NRGs between high-and low-risk groups in testing set (a), all set(b) and GEO set(c). Kaplan–Meier analysis of the survival probability between the two groups in testing set(d), all set(e) and GEO set(f). ROC curves to predict the sensitivity and specificity of 1-, 3- and 5-year survival according to the NRG_score in testing set(g), all set(h) and GEO set(i).

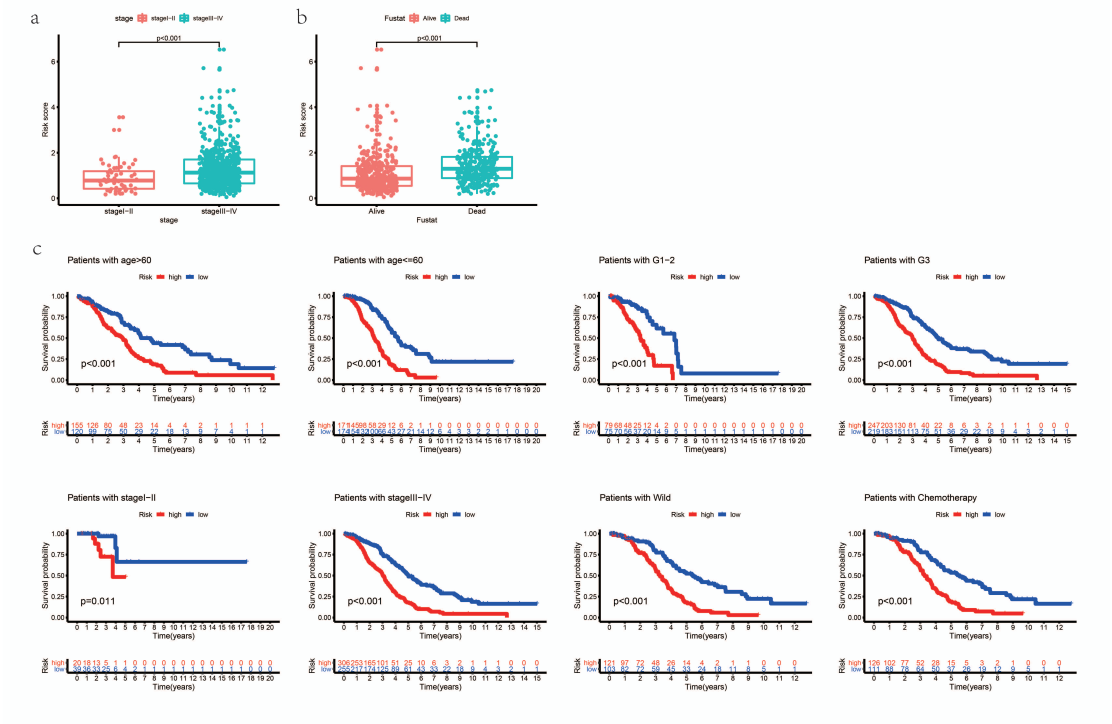

Fig S7 Clinical correlation analysis and stratified analysis of the model

Comparison of risk scores of different stages (a) and fustat (b). (c) Survival analysis of OV patients with different clinical characteristics.

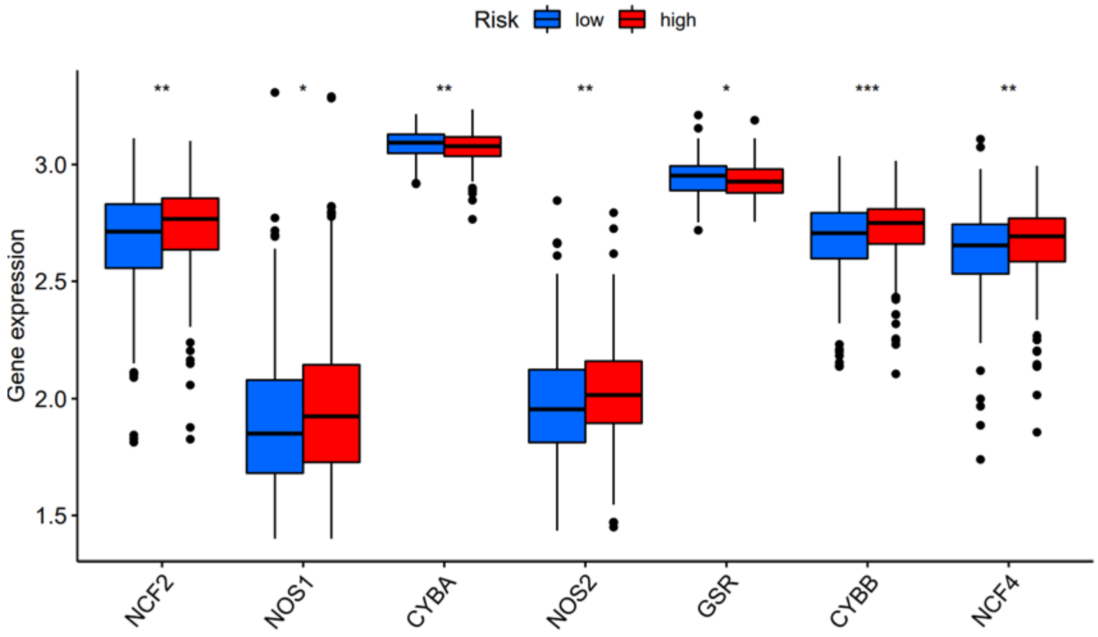

Fig S8 Expression levels of oxidative stress-related genes in high and low risk groups. *P<0.05, **P<0.01, ***P<0.001.

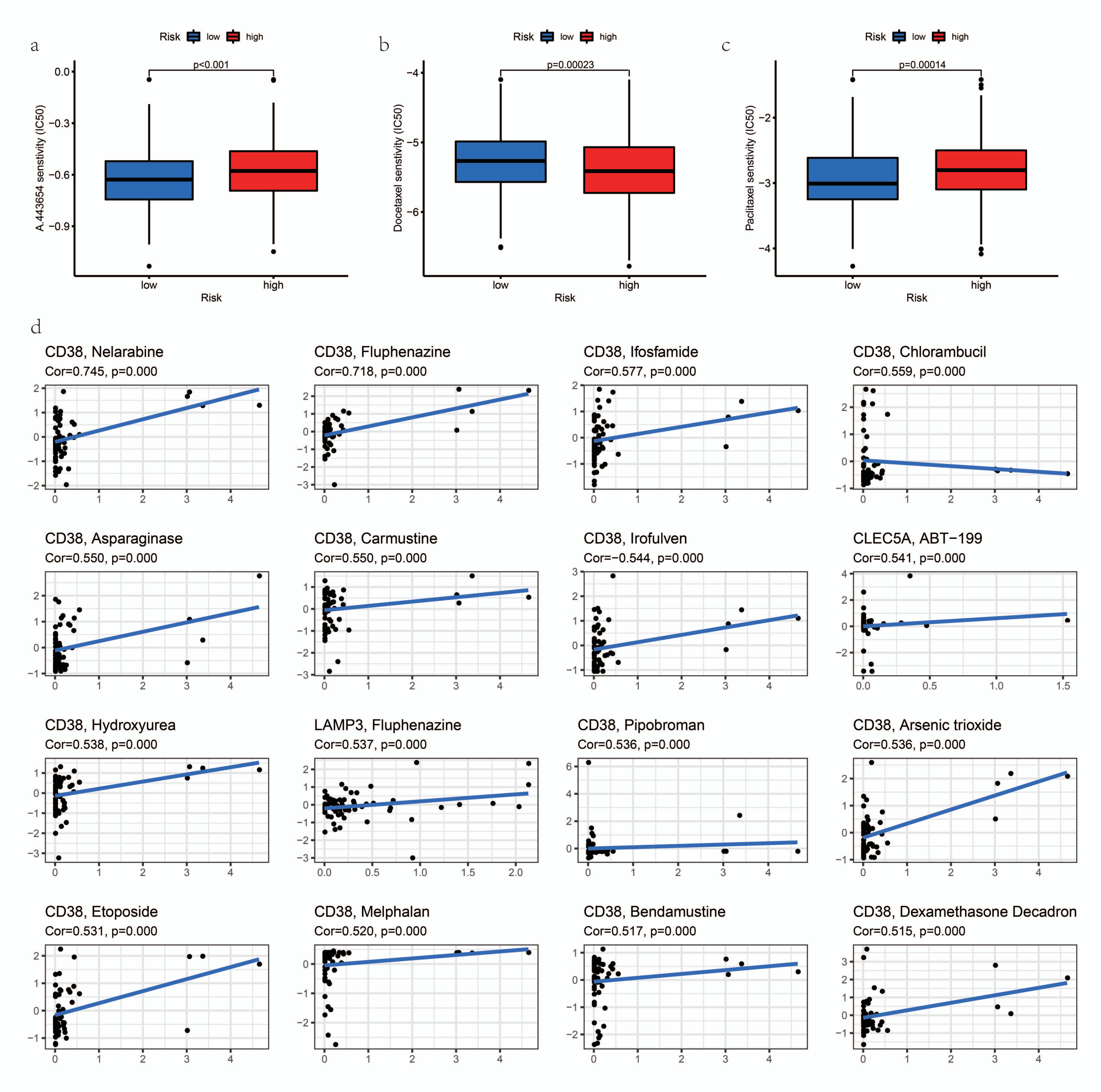

Fig S9 Analysis of drug sensitivity

The difference of IC50 of A.443654(a), docetaxel(b) and paclitaxel (c) in high and low risk groups. (d) Correlation between common drugs and 8 genes.

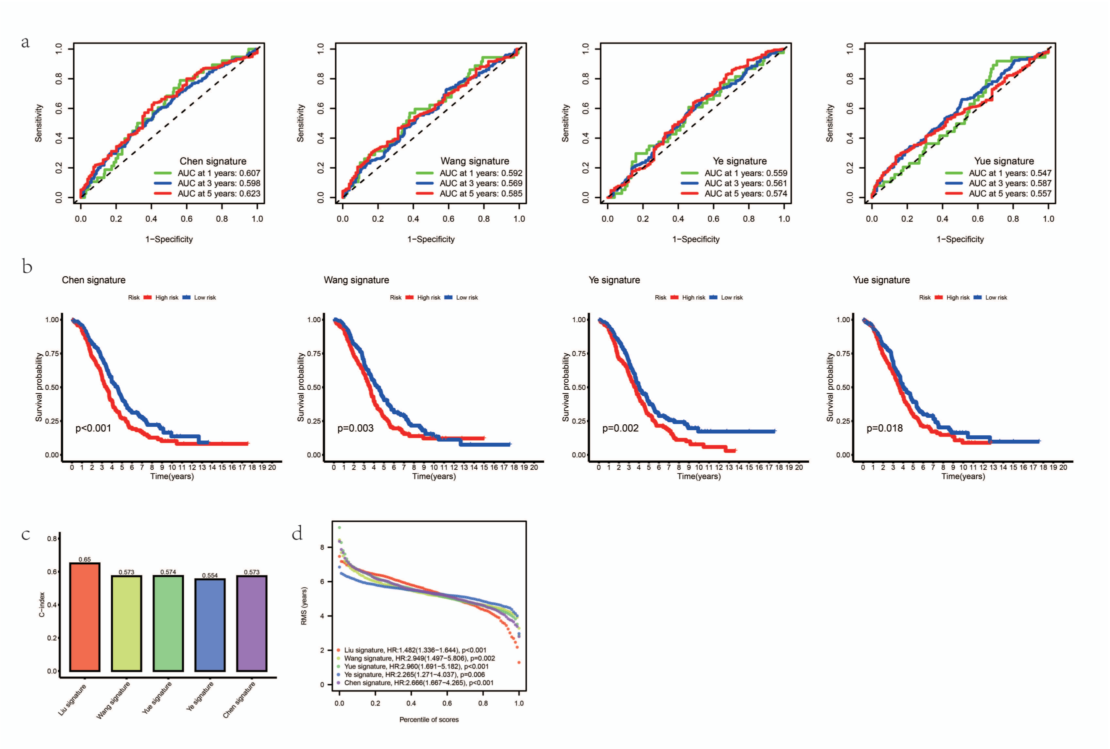

Fig S10 Comparison of our risk model with other established models

(a) ROC curves of four other published gene-signatures. (b) KM curves of four other published gene-signatures. (c) Concordance index (C-index) of the prognostic risk models. (d) Restricted mean survival (RMS) time curve of all five prognostic risk models.

Table S1 The clinical characteristics of OV patients in the TCGA and GEO databases.

Covariates	Type	Total
age	<=60	463(55.38%)
age	>60	473(44.62%)
grade	G1&G2	188(22.48%)
grade	G3	648(77.52%)
stage	StageI&II	84(10.05%)
stage	StageIII&IV	752(89.95%)
fustat	Alive	434(51.91%)
fustat	Dead	402(48.09%)
